# Follicular Dendritic Cell Sarcoma of Lymph Node: A report of a Patient with Chronic Myeloid Leukemia Treated with Imatinib

**Published:** 2015-07-01

**Authors:** Nisha Sharma, Ragini Singh, Nisha Marwah, Sumiti Gupta, Rajeev Sen

**Affiliations:** Department of Pathology, Pt. B. D. Sharma, PGIMS, Rohtak

**Keywords:** Follicular dendritic cell sarcoma, Chronic myeloid leukemia, Lymph node

## Abstract

Follicular dendritic cells or dendritic reticulum cells are important components of the immune system essential for antigen presentation. Malignancies arising from these cells are uncommon and the first case was reported in 1986. The most common sites of follicular dendritic cell sarcomas are lymph nodes, especially cervical, axillary and mediastinal regions, but extranodal sites including head and neck and gastrointestinal tract may be affected in one-third of patients. Immunohistochemistry plays an important role in its diagnosis to differentiate it from morphologically similar malignancies The present report describes a case of follicular dendritic cell sarcoma in a patient with chronic myeloid leukemia (CML) treated with imatininb mesylate for 6 years. This case deserves reporting due to rarity of the disease and hitherto unreported association with CML. Furthermore, the pathological diagnosis is challenging and requires a close-knit effort between the pathologist and haematologist.

## Introduction

 Follicular dendritic cell sarcoma (FDCS) is a rare neoplasm of follicular dendritic cells in lymphoid follicles. The disease usually involves the lymph nodes, especially the head and neck, mediastinal and axillary areas. Till now only approximately 150 cases have been reported worldwide.^1^ In recent years, there has been an increasing interest in this neoplasm due to availability of specific antibodies to confirm FDC lineage. The existence of this entity was first recognized by Monda et al. in 1986.^2^ FDCS was considered a low grade tumour with less tendency of recurrence or metastasis, but recent reports have revealed its more aggressive nature. FDCS is now considered as an intermediate-grade neoplasm. Here, we describe an extremely rare and previously unreported case of FDCS in a patient with chronic myeloid leukemia (CML) treated with imatinib.

## CASE REPORT

**1.2.** **Clinical presentation**

A 42-year-old female patient with chronic myeloid leukemia presented with complaints of progressively increasing abdominal distension since last 2 months and multiple bilateral lymph nodes in inguinal and axillary regions. The patient has been receiving imatinib mesylate for the past 6 years. The enlarged lymph nodes were first noticed by the patient 10 days prior to presentation. On examination, there was generalized lymphadenopathy and in the inguinal region enlarged lymph nodes were found, the largest measuring 2.5 cm. There was no hepatospleenomegaly. At the time of admission, hematologic findings showed mildly elevated total leukocyte count (12000/cumm), mild neutrophilia (neutrophil: 85%) and anemia (Hb: 7.5 gm/dl) with no blast in peripheral blood. Bone marrow findings showed trilineage hyperplasia with blasts<3%. Abdominal ultrasound revealed free fluid in pelvis. Moreover, computed tomography (CT) scan showed multiple enlarged peritoneal lymph nodes.

**1.2.2** **Pathological features**

 Histopathological examination of inguinal lymph node revealed a mass, measuring 2.5 x 2 x 1 cm. The cut section of lymph node was grey, white and fleshy. On microscopic examination, there was diffuse effacement of lymph node architecture by tumour cells arranged in fascicles and diffuse sheets (Figure 1a). The cells were spindle to ovoid scant to moderate cytoplasm and elongated to oval vesicular nuclei (Figure 1b). Small lymphocytes were scattered among the tumour cells. An occasional mitotic figure was noted. Reticulin stain showed expression around individual tumour cells (Figure 2). Possibilities of myeloid sarcoma, non- Hodgkin’s lymphoma, spindle cell carcinoma, malignant myeloma, gastrointestinal stromal tumour (GIST) and smooth muscle tumour were considered. 

**1.2.3** **Immunohistochemical findings**

Tumour cells were diffusely positive for CD 23 (Figure 3), CD 21 (Figure 4), CD35 and vimentin and showed variable positivity for CD 68 and S-100. CD 20 highlighted an occasional atrophic follicle separated by tumour cells (Figure 5). Small lymphocytes scattered among tumour cells were positive for CD 3. Tumour cells lacked Cytokeratin, HMB – 45, CD 30, Desmin, CD 117 and MPO. Based on the above findings, a final diagnosis of FDCS was made.

## Discussion

 Follicular dendritic cells, also known as dendritic reticulum cells, are a group of unique stromal cells which function as immune accessory cells in lymphoid and nonlymphoid organs.

These cells provide appropriate microenvironment within lymphoid tissue for antigen presentation to B cells to generate memory B cells and plasma cells.^1^^,^^3^

**Figure1a:**
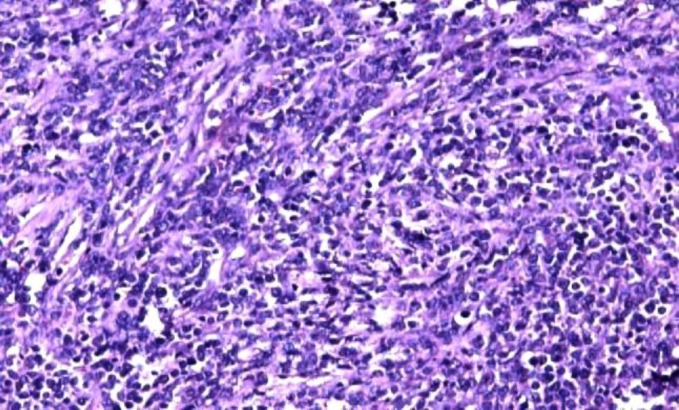
showing diffuse effacement of lymph node architecture by tumour cells arranged in fascicles and diffuse sheets, H&E (20x)

**Figure 1b:**
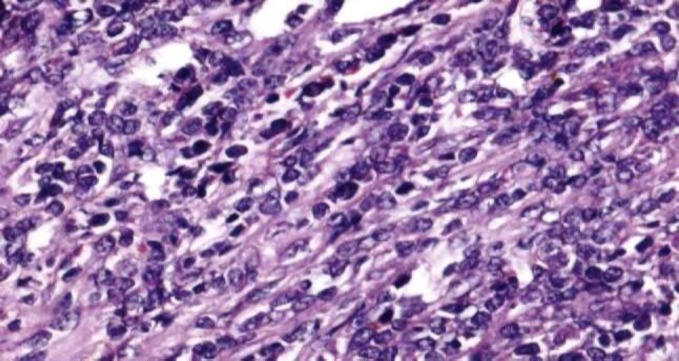
showing individual tumour cell morphology (spindle to ovoid scant to moderate cytoplasm and elongated to oval vesicular nuclei), H&E (40x)

**Figure2:**
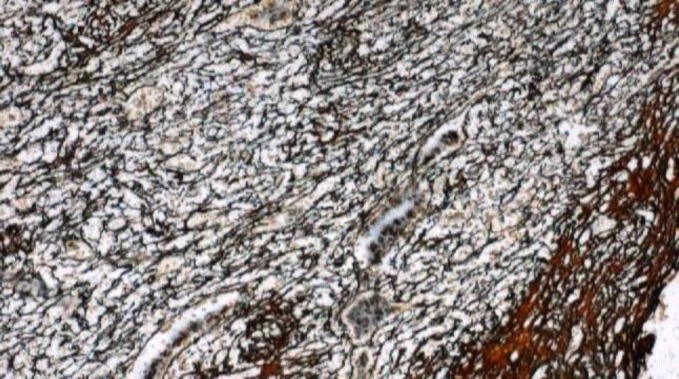
Reticulin stain showing expression around individual tumour cells (10 xs)

**Figure3:**
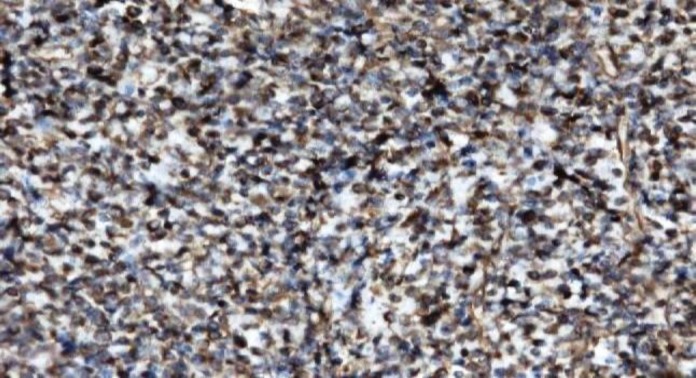
showing CD 23 positivity in tumour cells, IHC (20x)

**Figure 4:**
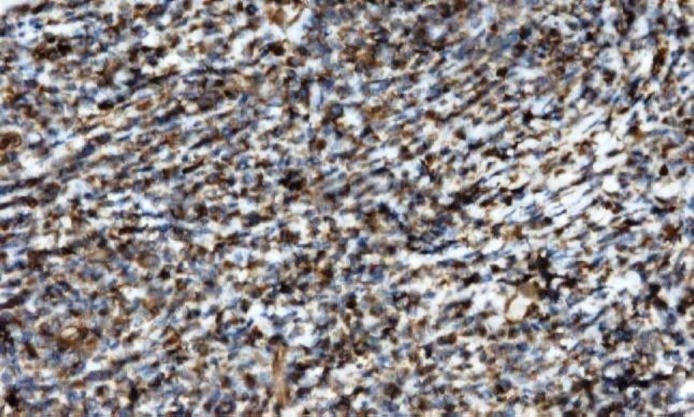
showing CD 21 positivity in tumour cells, IHC (20x)

**Figure 5:**
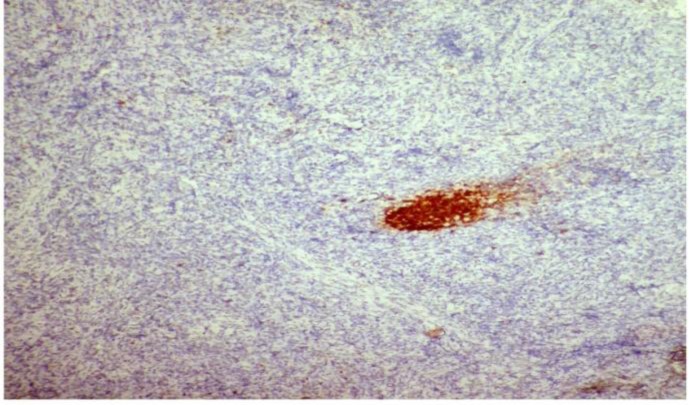
showing CD20 positivity in atrophic follicles, IHC (10x)

They have an important role in generation and regulation of germinal center reaction by prevention of apoptosis of germinal center B cells and by stimulating cellular reactions and proliferation.^4^ The cellular origin of FDCS is controversial with possible derivation from mesenchymal cells, hematopoeitic cells and recently suggested bone marrow stromal progenitor cells.^5^ Microscopically, tumour cells exhibit a storiform, fascicular, whorled, diffuse, follicle-like, or trabecular pattern. Neoplastic cell of FDCS is about one and half size of a macrophage, is ovoid to spindle shaped with elongated nuclei, irregular nuclear margins, small to medium distinct nucleoli and pale eosinophilic cytoplasm. Few cells show bi- or multinucleation. Mitosis is variable ranging from absent to 35/10 hpf.^6^

Clinically, this neoplasm has an equal sex predilection and occurs over a wide age range from 14-77 years (mean age: 47 years).^7^ The most common sites are cervical and mediastinal lymph nodes; however, one third of cases have been reported at extranodal sites like head and neck, soft tissue, breast, liver, spleen, skin and gastrointestinal tract. More aggressive behaviour by the tumour at intra-abdominal location has also been reported possibly due to late clinical presentation.^8^

Most of the information on FDCS is based on case reports or small case series, partly due to rarity of diagnosis and partly due to the under-recognition because of limited availability of more sensitive markers for FDC lineage. Case reports indicating its association with Castleman’s disease^9^ and Ebstein barr virus^10^ have been documented in literature; however, no definite etiology has been confirmed because of limited data.

The diagnosis of this tumour was challenging because of rarity of disease and unreported association with CML. Final diagnosis was only confirmed by IHC after possibilities of carcinoma (CK -, EMA -), lymphoma (LCA -, CD 3 -, CD20 -), malignant melanoma ( HMB 45 -), myeloid sarcoma (MPO -), smooth muscle tumour (Desmin -, SMA -) and gastrointestinal stromal tumour or GIST (CD117 -) were ruled out. CD 21, CD35 and CD 23 were performed which came out to be positive in tumour cells and finally diagnosis of FDC was made. Apart from these markers, other specific antibodies for FDC lineage are podoplanin and clusterin .^11^ The patient underwent 3 cycles of CHOP chemotherapy after diagnosis, but succumbed to her illness.

Because of the low incidence of this disease, it is difficult for any centre to acquire enough experience on appropriate management of these patients. With present treatment modalities, recurrence –free survival rate of 40% at 18-month follow-up and 27.4% at 5-year follow-up period has been reported.^6^ Although clear therapeutic guidelines are not available, surgical resection remain the mainstay of treatment with an undefined role of radiation and chemotherapy.^1^ Diagnosis in such a case depends on an array of morphologic, histologic, electron microscopic and most importantly IHC studies. A possibility of FDCS should be taken into account due to morphological resemblance once other lineage markers are reported negative.
